# Balancing science and political economy: Tobacco control and global health

**DOI:** 10.12688/wellcomeopenres.14362.1

**Published:** 2018-04-12

**Authors:** Mitsuru Mukaigawara, Janelle Winters, Genevie Fernandes, Devi Sridhar

**Affiliations:** 1 Global Health Governance Programme, Centre for Global Health Research, Usher Institute for Population Health Sciences and Informatics, University of Edinburgh, Edinburgh , UK; 2 Department of Medicine, Okinawa Chubu Hospital, Okinawa, Japan

**Keywords:** World Bank, World Health Organization, Tobacco, Framework convention on tobacco control, Bill and Melinda Gates Foundation, Bloomberg Philanthropies

## Abstract

**Background**: Global tobacco control is a major public health issue, as smoking-related disease burden remains high worldwide. The World Bank and the World Health Organization (WHO) are the driving forces in global tobacco control. However, little research has focused on their development, financing, decision-making, and accountability structures.

**Methods**: We used two strategies to identify the development and structure of global tobacco control initiatives. First, we reviewed the published literature through electronic databases. Second, we conducted grey literature searching.

**Results**: We identified four periods in the Bank’s involvement in global tobacco control, from creation of the evidence base in the 1990s to the implementation of tax reforms. We identified three phases in the WHO’s efforts, from its early recognition of the link between tobacco and health risks in the 1970s to its implementation of the Framework Convention on Tobacco Control. Both organisations are financed by a handful of private philanthropies, and face similar risks for effective tobacco control: reduced accountability and resource mobilisation, poor decision-making authority due to specific donor influence, and difficulty in monitoring and evaluation.

**Conclusions:** Continued attention should be paid not only to the primary health-related outcomes of tobacco use, but also to the decision-making and financing structures to promote tobacco control activities.

## Introduction

Tobacco use kills seven million people worldwide annually and tobacco-related disorders cause a substantial burden, accountable for approximately 150 million disability-adjusted life years ^1, 2^. Since the 1990s, evidence-based measures to combat tobacco use have been identified and implemented ^3^. At the global level, two international organisations, the World Bank and the World Health Organization (WHO), have become the most influential agencies in tobacco control. The Bank was the driving force in creating evidence on tobacco economics ^4^, and used its longstanding relationships with finance ministries to implement tax reforms in low- and middle-income countries ^3^. The WHO, the only normative agency in global health, used that evidence and negotiated toward the enactment of the Framework Convention on Tobacco Control (FCTC). At the same time as tobacco control became a cornerstone of the global health agenda, the past two decades also gave rise to philanthropic agencies, notably the Bill and Melinda Gates Foundation and Bloomberg Philanthropies. The Bank and WHO, given their resource limitations from governmental sources, have accepted this private support in pursuit of the tobacco control agenda.

The shift toward public-private partnerships for global tobacco control raises the risk of “Trojan multilateralism” ^5^, in which a handful of actors play significant roles in the decision-making processes of multilateral organisations. Safeguarding the Bank and WHO’s multilateral mandate and ability to make equitable decisions requires close attention to their governance ^6^. Analysis of global health projects, therefore, should include a broader scope of work, from primary health outcomes (e.g., mortality or the disease burden) to financing, decision-making, and monitoring structures ^7^. However, researchers have provided limited insight into the development, current decision-making, and financing structures of global tobacco control projects by the Bank and WHO ^8, 9^. The particular focus of previous research has been on creating epidemiological datasets and analysing FCTC negotiations ^2, 10^.

In this paper, we review published and grey literature on the Bank and WHO’s tobacco control policies. We use this literature to comparatively examine the development, current decision-making, and financial structures of each institution. We then identify major opportunities and challenges facing these two institutions in tobacco control priority setting and effective resource allocation.

## Methods

We used the following strategies to identify the development and structure of tobacco control established by the WHO and the Bank. First, we reviewed the published literature ( Supplementary File 1). We primarily focused on the governance and financing structures of each organisation. Therefore, literature that focused on general cost-effective measures and epidemiological studies were excluded. Second, we searched for research and project reports through the websites of the Bank (Projects and Operations database, Development Topics database, Documents and Reports database, digitalised archive holdings, annual trust funds reports, and Finances trust fund paid-in contributions database), WHO, Organization for Economic Co-operation and Development (OECD), Gates Foundation, Bloomberg Philanthropies, International Monetary Fund (IMF), and Institute for Health Metrics and Evaluation (IHME).

## Results

### Tobacco control and the World Bank

**Figure 1.**
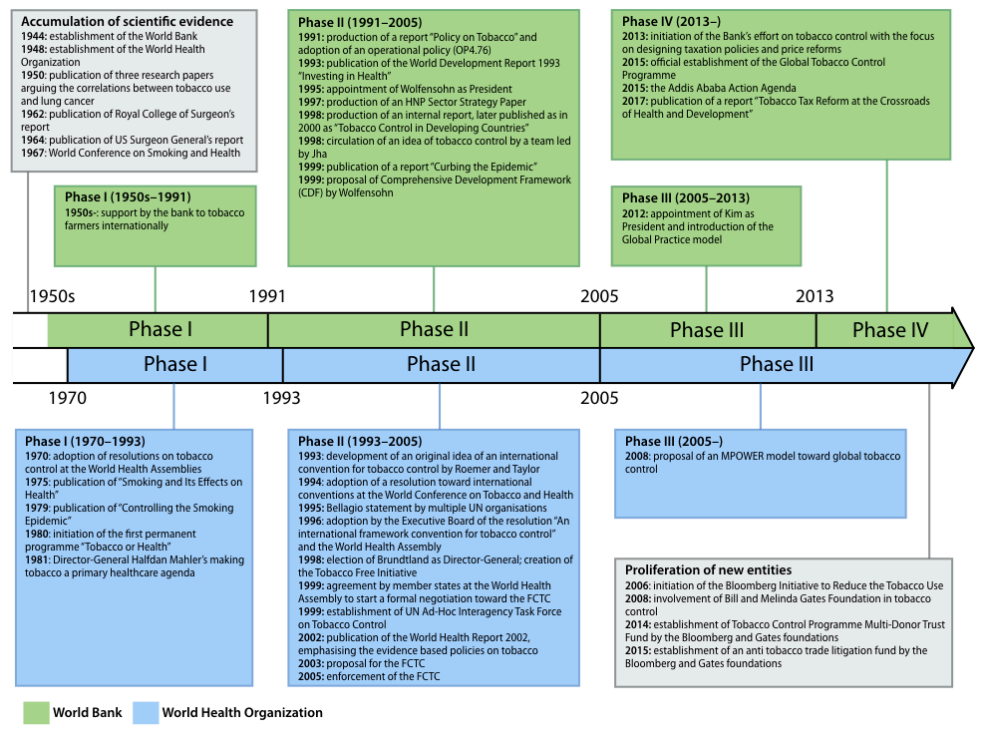
Timeline of key events in tobacco control by the World Bank and the World Health Organization (WHO). The World Bank’s tobacco control programmes can be captured in four phases, from its support to tobacco production in the 1950s to the implementation of tax reforms in the 2010s. The WHO recognised the tobacco’s harm early, and its efforts are divided into three phases.

Evidence of tobacco’s danger first became available in 1950, with the publication of three research papers, which reported a correlation between tobacco consumption and lung cancer ^11^. The initial evidence was followed by reports from the Royal College of Physicians of the United Kingdom (1962) ^12^ and the United States Surgeon General (1964) ^13^. This accumulated evidence also paved the way for the first international tobacco control event, the World Conference on Smoking and Health, in 1967. However, it took several decades for the Bank to initiate tobacco control projects worldwide. We identified four major periods in the Bank’s involvement in global tobacco control ( Figure 1).

***Phase I: The politicisation of tobacco control (1950s–1991).*** Starting in the 1950s, the Bank extensively supported tobacco farmers, as part of an effort to strengthen economies by increasing specific countries’ exports of expensive crops ^14^. According to the Bank’s Projects & Operations database, the Bank committed USD 32 million in the 1970s to four projects aimed at enhancing tobacco production in Tanzania, Zambia, and Uganda ^15– 18^. However, in 1991, the Bank adopted an Operational Policy prohibiting it to “lend directly for, invest in, or guarantee investments or loans for tobacco production, processing, or marketing” (See World Bank Operations Manual).

This reversal of tobacco lending policies was largely due to the efforts of a handful of the Bank staff in the early 1990s. Howard Barnum, a then senior economist, played a key role in demonstrating the cost-effectiveness of tobacco control ^19^. A tension existed between the humanitarian concept of health as a human right, particularly following the Alma Ata declaration of 1978 ^20^, and the Bank’s economically-driven, politicised development approach to health ^21^. To prioritize investment in tobacco control for health purposes, therefore, the Bank needed to demonstrate its cost-effectiveness. Senior economist Howard Barnum advanced a particularly compelling argument that market efficiency could not be applied to tobacco because of its addictiveness and consumers’ lack of knowledge of its dangers ^19^.

***Phase II: Creation of the knowledge bank (1991–2005).*** The Bank then emerged as a “knowledge bank”, which could create and marshal expertise on development topics ^22^. In the early 1990s, the Bank still saw tobacco as a health-versus-economics issue; the 1993 World Development Report argued for the importance of tobacco taxation, but simultaneously urged caution in applying tax reforms in countries that relied on tobacco exports ^23^.

With the accumulation of evidence on tobacco’s dangers, the Bank began to make tobacco a political agenda in the mid-1990s. The 1997 Health, Nutrition and Population (HNP) Strategy Paper stated that tobacco control required a cost-effective approach, such as taxation or price measures. Based on this paper, researchers in the Bank’s HNP sector, led by Prabhat Jha, presented the idea of tobacco control to the HNP board in 1998 ^24^. They proposed potential control activities, including the production of an analytic report, construction of partnerships with other organisations, and the support for the WHO’s efforts. In this period, President James Wolfensohn also proposed a Comprehensive Development Framework, in which he noted that development required a holistic approach; health was central to the development; and tobacco control was a key development agenda ^25^.

There was opposition to this control agenda inside the Bank. Some argued that the policy was against the idea of trade liberalisation. One needs to note that the Uruguay Round was concluded around this time, which enhanced trade liberalization and led to the establishment of the World Trade Organization. Thanks to Barnum’s efforts, however, the Bank had already adopted its 1991 operational policy, which forbade its investment in tobacco production. Despite struggling with this opposition, the Bank continued its effort to create the evidence for tobacco control. Such efforts were crystallised in the publication of two reports on the economics of tobacco-related deaths ^4^ and its evidence-base in developing countries ^26^.

This second phase was also characterised by a close collaboration with the WHO. Gro Harlem Brundtland was elected as the WHO’s Director-General, and initiated the Tobacco Free Initiative in 1998. The WHO’s leadership enhanced inter-agency efforts in tobacco control, including the Bank’s tobacco control projects.

***Phase III: Proliferation of new actors in tobacco control (2005–2013).*** The Bank provided the WHO with the evidence-base on tobacco’s harm and the cost-effectiveness of tobacco control, which became a driving force toward the enactment of the FCTC in 2005. However, after its enactment, the Bank entered a slow phase in tobacco control. Some researchers speculate it was because of Bank bureaucracy; taxation and health were in different silos and the two policy groups could not collaborate with each other effectively on implementing tax reforms (See Devex article on The World Bank and Tobacco Taxes). Another possible explanation is the lack of leadership for tobacco control. Following President James D. Wolfensohn’s retirement in 2005, the new President Paul Wolfowitz (1995–1997), showed little interest in global health ^27^.

In this phase, new actors started to work on tobacco control globally, including the Gates Foundation and Bloomberg Philanthropies. In 2006, the Bloomberg Philanthropies initiated the Bloomberg Initiative to Reduce the Tobacco Use in developing countries, based on the success in tobacco control in New York City. In 2008, this initiative went into the second phase, and was joined by the Gates Foundation (See Gates Foundation press release).

***Phase IV: Global Tobacco Control Programme (2013–).*** The Bank launched multiple country-based tobacco control projects that were initiated around 2013 (Development Topic database). In 2015, the Bank officially initiated the Global Tobacco Control Programme, which enhanced collaborations between the Bank’s HNP Global Practice and its Global Taxation Team. Such efforts were summarised in its major 2017 report, *Tobacco Tax Reform: At the Crossroads of Health and Development* ^3^.

The major driver for renewed efforts in tobacco control at the Bank was the election of President Jim Yong Kim and effective domestic resource mobilisation for health. President Kim introduced a new model of Global Practices, and tobacco control was placed in the HNP Global Practice, which fostered interactions between technical staff working in different regions. Also, in order to implement tax reforms in target countries, the Bank needed to establish buy-ins from country experts and leaders. The Bank therefore began to collaborate closely on tax reforms with experts from the Ministries of Finance in these countries. The results from such collaborations have been published in multiple Bank country reports ^3^.

**Figure 2.**
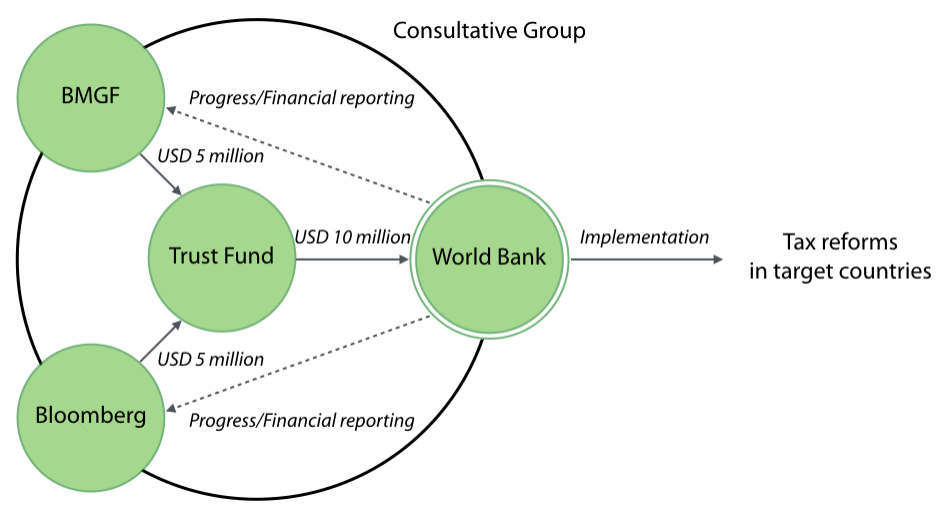
Governance structure of the World Bank’s tobacco control programme. The World Bank’s Global Tobacco Control Programme is funded by a trust fund, which is financed by the Gates Foundation and the Bloomberg Philanthropies. The World Bank has implemented tax reforms in target countries since 2013. The decision-making authority is named the Consultative Group, which is chaired by the World Bank and participated by the representatives of the two donors. Progress reports and financial information are available on a secure website for the two donors. Abbreviations: BMGF: Bill and Melinda Gates Foundation, Bloomberg: Bloomberg Philanthropies. Sources: 29, 30.

The Global Tobacco Control Programme is financed through the Tobacco Control Programme Multi-Donor Trust Fund, supported by the Gates Foundation and the Bloomberg Philanthropies ( Figure 2). Bloomberg Philanthropies and the Gates Foundation each committed USD 5 million to the three-year (December 2014 to 2017) fund. It is a bank-executed trust fund; this type of trust fund typically supports advisory services and the development of the Bank’s knowledge agenda ^28^. According to the trust fund agreement, the supported projects have a narrow objective to assist selected countries in implementing tobacco tax reforms ^29, 30^. These objectives are accomplished through four major tobacco control activities: providing ministries with policy advice and technical assistance; establishing knowledge exchange systems; helping to build capacity and promote tobacco control as a priority; and coordinating the programme with appropriate partnership. The fund is governed by a Consultative Group, which is chaired by the Bank, and includes representatives of the donors, the Gates Foundation and Bloomberg Philanthropies. This Consultative Group selects country programmes.

### Tobacco control and the WHO

The development of tobacco control frameworks at the Bank and WHO shared several features: both organisations faced internal objections, identified collaborations within the UN agencies as a key to success, and had strong leadership, from Wolfenson and Brundtland, respectively. In contrast to the Bank, however, the WHO recognised the scientific evidence early on, and subsequently initiated its control efforts in the 1970s. ( Figure 1)

***Phase I: Tobacco control as a primary healthcare issue (1970–1993).*** In 1970, the WHO adopted its first tobacco resolution, which urged the Director-General to consider establishing an expert group on tobacco control. The resolution also highlighted the importance of educating younger generations on tobacco’s harm ^31^. Such efforts were advanced through two landmark reports: *Smoking and Its Effects on Health* in 1975, and *Controlling the Smoking Epidemic* in 1979. The 1979 report called for action ^32^, based on which the first permanent programme “Tobacco or Health” was created in 1980 ^33^. Although it started small, this programme contributed to the preparation of technical reports ^34^ and lead to the inception of the World No Tobacco Day ^35^ to raise awareness among member states. This period coincided with the organisation’s programming goal “Health for All,” which aimed to secure the health of all people through primary healthcare. Director-General Halfdan Mahler integrated tobacco control in a primary healthcare agenda ^36^.

***Phase II: Toward enactment of the FCTC (1993–2005).*** In the 1980s, some researchers expressed their support for an international law to regulate the consumption of tobacco products ^37^. However, it was in the 1990s when the WHO developed a realistic idea for a global tobacco control facility. The original idea, discussed by lawyers Ruth Roemer and Allyn Taylor in 1993, was to use the WHO’s constitutional authorities ^38^. Despite opposition inside and outside of the WHO ^38^, the World Health Assembly adopted a resolution in 1995 to develop an international framework on tobacco control. After Brundtland’s election in 1998, the idea gained political support, and the Tobacco Free Initiative (TFI) was launched ^39^.

Tobacco control was different from other health problems by nature, due to active industry lobbying. The WHO’s noteworthy strategy was to gain support from the entire UN system ^39^. Brundtland requested that other organisations shift the leadership role in tobacco control to the WHO. The WHO became the coordinator of the UN and Bretton Woods systems, leading to the creation of an *ad hoc* Inter-Agency Task Force in 1999. In that year’s World Health Assembly, member states agreed to start a formal negotiation toward the proposed framework ^40^. Beyond paving the way to the FCTC in 2005, Brundtland’s move created a momentum across the UN system. For example, in the Inter-Agency Task Force meeting, the UN linked tobacco to the eight Millennium Development Goals (MDGs) ^41^, which pushed tobacco control into a global development agenda ^42^.

***Phase III: Implementation of the FCTC (2005–).*** After the enactment of the FCTC, the WHO implemented tobacco control projects globally. The FCTC serves as a negotiation entity for resource allocation and decision-making, whereas the TFI conducted technical support for national or regional tobacco control activities, with the support of Bloomberg Philanthropies.

**Figure 3.**
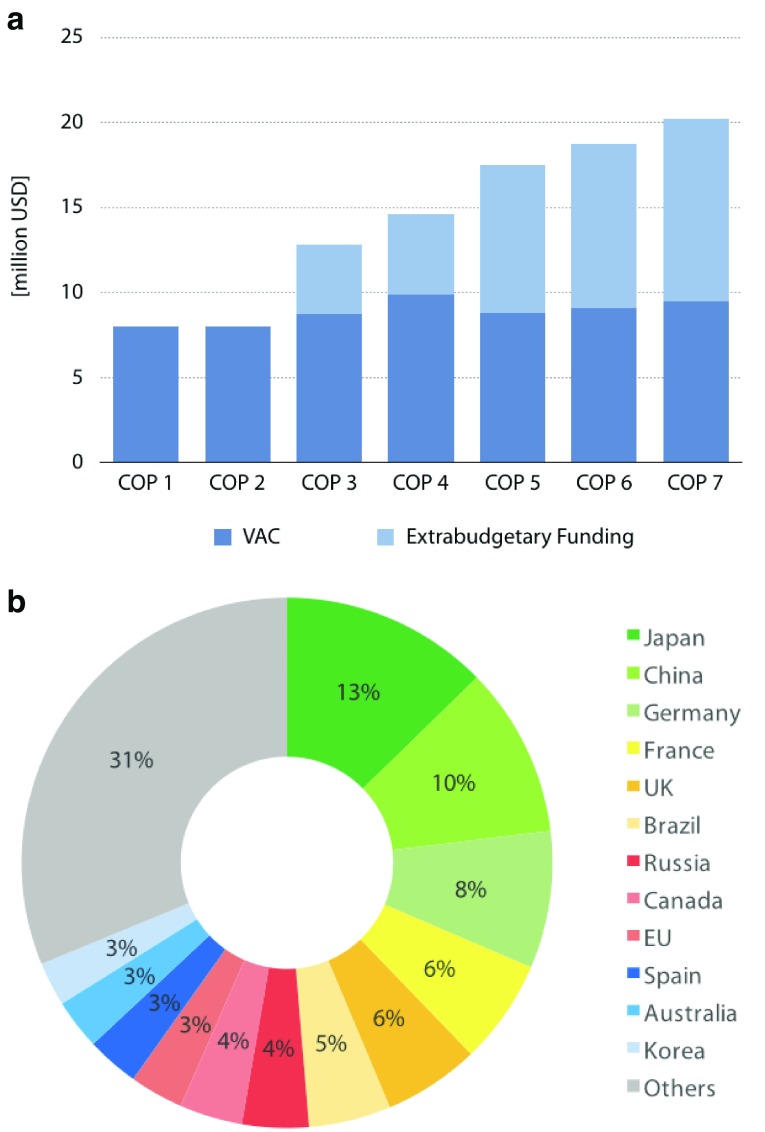
Budget of the World Health Organization’s (WHO) FCTC. ( **a**) Trends of voluntary assessed contributions (VAC) and extra-budgetary funding [USD], 2006–2019. The WHO’s FCTC is financed by the VAC and extra-budgetary funding. The FCTC was initially financed solely by the VAC, but now the extra-budgetary funding exceeds the VAC. ( **b**) Voluntary assessed contributions (VAC) for years 2018–2019. The major contributor to the VAC includes Japan, China, Germany, France and United Kingdom. Abbreviations: COP = Conference of Parties; EU = European Union; UK = United Kingdom; VAC = Voluntary assessed contributions. Source: http://www.who.int/fctc/en/.

The biannual Conference of the Parties (COP) is the FCTC’s decision-making entity. The COP is attended by the Parties (181 countries as of March 2018), and discusses its budget and programmes proposed by the Secretariat. The main sources of the funding ( Figure 3a) are the Voluntary Assessed Contributions (VAC) ( Figure 3b) by the Parties and extra-budgetary funds. Therefore, the FCTC is essentially financed by WHO trust funds: resources mobilised voluntarily from donors, and held apart from the core budget ^28^. Sources of extra-budgetary funds include governmental and non-governmental institutions such as the European Union ^43^ and the Gates Foundation ^44^.

**Figure 4.**
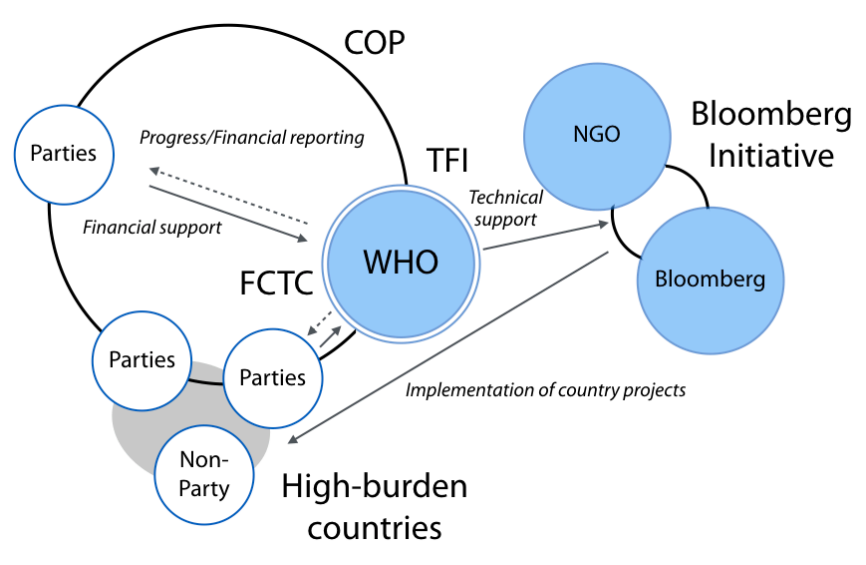
Governance structure of the World Health Organization’s (WHO) tobacco control programme. The WHO functions as the secretariat of the FCTC and the TFI. The FCTC is funded by the voluntary assessed contributions and extra-budgetary funding, the former from the Parties of the FCTC. The WHO is accountable to the Parties, and documents are available on its public website. The TFI produces technical reports, but its country projects are implemented as the Bloomberg Initiative to Reduce Tobacco Use. The WHO is a part of this initiative, but has no authority in the selection of funded projects. Abbreviations: Bloomberg = Bloomberg Philanthropies; COP = Conference of Parties; FCTC = Framework Convention on Tobacco Control; NGO = Non-governmental organisations; TFI = Tobacco Free Initiative. Sources: http://www.who.int/fctc/en/, http://www.who.int/tobacco/about/partners/bloomberg/en/.

The TFI supports national and regional tobacco control programmes. With the financing and close collaboration of Bloomberg Philanthropies, the WHO’s TFI works with countries as part of the Bloomberg Initiative to Reduce Tobacco Use. The exact amount of funding is not available on public websites, but the initiative has provided grants to implement tobacco control with a focus on high-burden countries. The WHO, however, does not play any role in the grant’s selection process ( Figure 4) (See WHO page on TFI).

### Challenges and opportunities of the World Bank and WHO

***Financing, decision-making, and accountability of tobacco control.*** A comparative analysis of the tobacco control policies by the Bank and WHO illustrates similarities and differences in their financing, decision making, and accountability ( Table 1). First, both institutions rely on a trust-fund-like model. The Bank’s projects are supported by a multi-donor trust fund by the Gates Foundation and Bloomberg Philanthropies. The WHO’s FCTC is implemented mostly by the VAC and extra-budgetary funding, which are all voluntary funding sources.

**Table 1.** Origin and the current status of tobacco control by the World Bank and World Health Organization.

	World Bank	World Health Organization
Response to scientific evidence	1990s	1970s
Major success	Identification of tobacco control as a market failure	Mobilising solidarity toward the enactment of FCTC
Approach	Economics driven	Political, diplomatic
Current entity of implementation	Global Tobacco Control Programme (2015–)	FCTC (2005-) Tobacco Free Initiative (TFI) (1998–)
Objectives	To assist countries in designing tobacco tax reforms	To tackle causes of tobacco epidemic: trade liberalization, direct foreign investment, tobacco advertising, promotion and sponsorship, and illicit trade
Location of activities	Low- and middle- income countries	Parties (181 as of November 2017), but the TFI works particularly in high-burden countries
Major source of funding	Trust fund model: Multi-donor trust fund financed by the Gates Foundation and Bloomberg Philanthropies	Trust fund model: voluntary assessed contribution by Parties, supplemented by extra-budgetary funding
Budget	USD 6,906,000 in two years (2014–2015)	USD 17,470,000 in two years (2014–2015)
Governance	Consultative Group, with representation of the Bank (Chair) and representatives of donor agencies	FCTC: Conference of Parties (COP), held biannually, with the Parties and observer participation TFI: WHO is not involved in the selection process of the Bloomberg grants
Accountability	Accountable to donors (detailed progress reports available in the secure website)	Accountable to the Parties (COP official document available on the public website)

Second, in terms of decision-making processes, the FCTC is the only entity that is not influenced by private philanthropies. The FCTC holds biannual COPs, in which the Parties (sovereign states) have the voting power. In contrast, the WHO’s TFI is not involved in the selection process of country projects supported by Bloomberg Philanthropies’ tobacco control grants. Also, the Bank holds a Consultative Group, which includes the representatives from the Bank, the Gates Foundation, and Bloomberg Philanthropies.

Third, the Bank is accountable mainly to its two donors funding tobacco control, and detailed progress reports and financial disclosures are available only for these donors on its secure websites ^29, 30^. The WHO, on the other hand, is accountable mostly to its Parties, but most of the documents from the COP are disclosed on its website. Such detailed financing data are not available for the WHO’s TFI.

**Figure 5.**
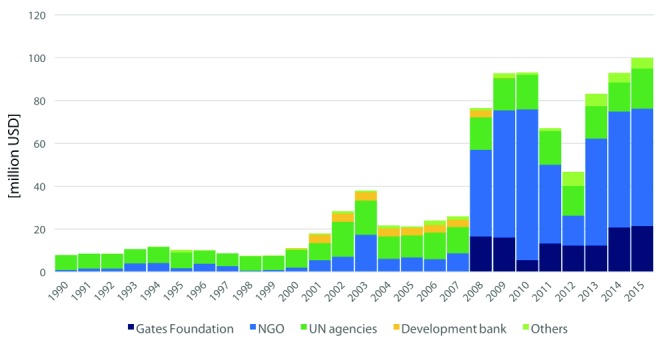
Development assistance for health (DAH) for tobacco control by channel of assistance [million USD] Abbreviations: Gates Foundation = Bill and Melinda Gates Foundation; NGO = Non-governmental organisations; UN = United Nations. Source: https://vizhub.healthdata.org/fgh/

***Challenges and opportunities.*** What are the opportunities that the two organizations created for tobacco control? The FCTC provides an international platform for financial and legal support to tobacco control activities. Through the FCTC, Parties can negotiate and support global efforts, and civil societies can participate in the conferences as observers. The Bank has also created the evidence on tobacco control, and implemented tax reforms globally. Based on this evidence base, attention is now being paid to effective implementations of tobacco control measures. Finally, funding has increased for tobacco control globally, reaching from USD 7.9 million in 1990 to USD 99.8 million in 2015 ( Figure 5) (See Viz Hub page on financing global health). There is a growing diversity in channels of global financial assistance, such as nation states, philanthropies, and NGOs. Such coherence toward tobacco control was not observed in the early 1990s, when the amount of funding was limited and mostly channelled by the UN organisations.

However, such opportunities potentially pose challenges as well. As leaders in tobacco control, the two organisations should consider three issues: their institutional segmented nature, the proliferation of new actors, and monitoring and evaluation.

First, tobacco control will continue to be challenged by the segmented nature of global health institutions and government ministries as well as global regime complexity, i.e., conflict between trade and health law ^45^. An initiative can easily be classified into one sector, to which other sectors pay little attention. Some argued that this occurred in the late 2000s to early 2010s in the Bank, during which tobacco control was implemented only by health experts, and did not collaborate effectively with fiscal policy groups to implement tax reforms globally (See Devex article on The World Bank and Tobacco Taxes). Such miscommunication across sectors could occur in national governments as well. The Bank has made enormous efforts to build national capacities in implementing tobacco taxation since 2013, but the existing bureaucracies across ministries should be scrutinised; implementation, monitoring and evaluation of taxation measures require collaborations that go beyond the authority of finance and health ministries.

In addition, tobacco industries are undertaking a growing number of tobacco control litigations under trade law ^46^. Tackling such litigations requires close collaboration between health and legal practitioners, which poses a particular challenge for the FCTC, since it cannot legally force the Parties to adhere to the provisions ^47^.

Second, the proliferation of donors, such as the Gates Foundation, is a double-edged sword. Tobacco control programmes are not the exception to the shift in global health governance, in which multiple actors – including nation states, philanthropies, NGOs, and industries – are involved in decision-making. The involvement of private philanthropies poses risks, such as the alignment of objectives of implementation bodies and their own, and the use of material incentives ^5^. For example, in 2010 the Gates Foundation terminated a tobacco control grant to a Canadian agency, the International Development Research Centre, because the leadership of the organisation also directed Canadian tobacco industries (see Gates Foundation press release). Also, the Bank’s efforts are currently implemented with a narrow mandate that matches the philanthropies’ objectives: tax reforms in targeted countries. Similarly, the WHO’s TFI operates with a particular focus on high-burden countries without having any governmental authorities in the grant’s selection process. Such an approach possesses an inherent risk of “Trojan multilateralism”, the ability of a few agents (such as specific states or industry representatives) to have an undue influence on decision-making at multilateral organisations ^4^.

Lastly, monitoring and evaluation have become increasingly difficult due to the trust funds’ inadequate transparency ^28^. Tracking development assistance for health (DAH) allows researchers to analyse the trend of priorities set by donors and implementing agencies, and the lack of a comprehensive database to track development assistance for trust funds has been a major concern. Although the Bank sometimes releases annual trust reports, they are not available for recent years, and its Trust Fund Directory is out of date. Furthermore, its World Bank Finances database’s trust funds datasets generally only include trust fund commitments after 2005, and were last updated in 2013–2014 ^28^. Datasets available from other institutions such as the IHME, AidFlows (a collaboration between the Bank, OECD, and regional development banks), and the OECD also do not capture the entire picture of the trust funds. For example, our literature search identified at least seven trust funds for tobacco control projects that were channelled by the Bank ( Table 2), but no databases systematically included all these activities. Moreover, while the IHME and AidFlows allow researchers to specifically search for Gates Foundation commitments, Bloomberg Philanthropies’ contributions were either unavailable (AidFlows) or included in NGO totals (IHME). Given that the Bloomberg Philanthropies has invested approximately USD 1 billion in tobacco control (see Bloomberg page on tobacco control), this is a concerning trend. The health financing landscape for tobacco control may look significantly different, if such activities are properly reflected and private foundation commitments are clearly identifiable.

**Table 2.** Examples of trust fund for tobacco control channelled by the World Bank.

Year	Donor	Trustee and Trust fund ID	Amount (USD)	Source
2005	World Health Organization	Global Tobacco Control Activities (TFM22664)	200,000	WB Finances, AidFlows
2005	World Health Organization	Report on Economics of Tobacco Control (TFM23338)	308,000	WB Finances, AidFlows
2005	US Department of Health and Human Services	Stronger Tobacco Control within a Sound Economic and Social Framework (TFM24236)	529,000	WB Finances, AidFlows
2005	World Health Organization	WHO Grant for Protecting Youth from Tobacco in 5 countries (TFM24334)	183,985	WB Finances, AidFlows
2015	Swiss Agency for Development and Cooperation	Reducing Health Risk Factors in Bosnia and Herzegovina	1,100,000	AidFlows
2014–17	Bill and Melinda Gates Foundation	Tobacco Control Program Multi-Donor Trust Fund (TF072332)	5,000,000	Official trust fund documents, AidFlows
2014–17	Bloomberg Philanthropies	Tobacco Control Program Multi-Donor Trust Fund (TF072332)	5,000,000	Official trust fund documents

The World Bank Finance’s dataset on paid-in contributions to trust funds includes a disproportionate number of trust funds in 2005. 2005 is the first year included in the dataset, and many of these trust funds may therefore be mislabelled as 2005 commitments. The WHO Grant for Protecting Youth from Tobacco in 5 Countries, for example, is more likely to be from 2001. Sources: 29, 30, 48, https://data.worldbank.org/data-catalog/m54j-ersw.

## Conclusions

Despite the small beginnings in the 1970s, the past two decades have seen the crystallisation of global efforts in tobacco control by the Bank and the WHO. This has resulted in major progress through the establishment of the FCTC and the implementation of evidence-based tobacco control policies with a particular focus on low- and middle-income countries.

The Bank and the WHO are the driving forces for tackling the global epidemic of tobacco smoking, given their normative functions, influence, and ability to catalyse collaborations. Attention should be paid to the inherent risks of current governance structures: the segmented nature of institutions and ministries, concentration of funding from philanthropic institutions, and inadequate transparency on the trust fund funding flows and activities. Further research is necessary to identify each donor’s financial contributions to the Bank and the WHO. Independent, public monitoring processes of the WHO’s TFI and the Bank’s tobacco control efforts should also be considered.

## Data availability

Data for this article are available on Open Science Framework. Dataset 1: Global tobacco control and the World Bank/WHO. http://doi.org/10.17605/OSF.IO/PH7NM ^49^

Data are available under the terms of the Creative Commons Zero "No rights reserved" data waiver (CC0 1.0 Public domain dedication).

## Supplementary material

Supplementary File 1 – Literature searching methods.
