# Supplementary material for: Balancing science and political economy: Tobacco control and global health
Source: Wellcome Open Res. 2018 Apr 12;3:40. [Version 1] doi: 10.12688/wellcomeopenres.14362.1 (PMC6097416; doi:10.12688/wellcomeopenres.14362.1)

**Supplementary File 1. Literature searching methods**

The search strategy yielded 11,844 results (figure), of which 4,328 were duplicates and removed. The databases included Medline (<https://www.ncbi.nlm.nih.gov/pubmed/>), Web of Science (<http://www.webofknowledge.com/>), Embase (<https://www.embase.com/>), LILACS (<http://lilacs.bvsalud.org/en/>), POPLINE (<https://www.popline.org/>), and Cochrane Library (<http://www.cochranelibrary.com/>). We used the following combination of keywords: tobacco, smoking, cigarette, World Bank, International Bank for Reconstruction and Development, International Development Association, World Health Organization, and Framework convention on tobacco control. For example, the Medline database was searched as follows: (“tobacco” OR “smoking” OR “smoker*” OR “cigar*” OR “cigarette*”) AND ((“World Bank” OR “International Bank for Reconstruction and Development” OR “International Development Association”) OR (“World Health Organization” OR “Framework convention on tobacco control” OR “FCTC”)).

Literature that aimed to describe smoking prevalence in general, or cost-effectiveness analyses of tobacco control measures were excluded. Screening the papers revealed 129 which provisionally met the criteria based on their abstracts. After the assessment of their full-texts (n=129), 69 papers were analysed and included in the analysis. Among the analysed 69 literature, studies that cited the same sources were excluded.


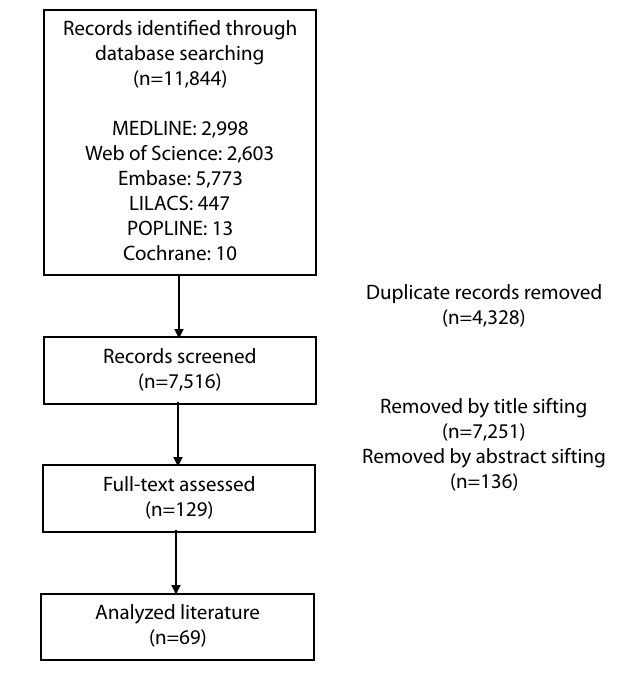

Supplement: Supplementary file 1 [file wellcomeopenres-3-15626-s0000.tgz › 0a7e72ba-4776-4831-bd3b-1f45fed9647e.docx]
