# Supplementary material for: Balancing science and political economy: Tobacco control and global health
Source: Wellcome Open Res. 2018 Apr 12;3:40. [Version 1] doi: 10.12688/wellcomeopenres.14362.1 (PMC6097416; doi:10.12688/wellcomeopenres.14362.1)
Supplement: Peer review history

# Peer review: Referee response for version 1

**DOI:** 10.21956/wellcomeopenres.15626.r33113

**Published:** 2018-07-03

**Authors:** Jappe Eckhardt

**Affiliations:** 1 Department of Politics, University of York, York, UK

This article by Mukaigawara, Winters, Fernandes  and Sridhar is a welcome contribution to the existing tobacco (control) literature, which has focused primarily on the health-related outcomes of tobacco use. Given that, as the authors note, “smoking-related disease burden remains high worldwide”, continuous attention for health related issues is of course of utmost importance. Yet there is also a need to better understand the political economy of tobacco control and that is one of the key aims of this article by Mukaigawara *et al.*

 The author assess the role of the two international organizations (IOs) that have arguably played the most important role in global tobacco control: the WHO and the World Bank. The authors provide an interesting historical overview of their development, the way both IOs finance their tobacco control efforts, as well as their governance- and accountability structures. The article shows how both organisations have been instrumental in the development of the establishment of Framework Convention on Tobacco Control (FCTC) and implementing evidence-based tobacco control policies across the globe. Yet, the authors also point at some important weaknesses in the governance structures of the WHO and the World Bank when it comes to their tobacco control efforts. One of the key weaknesses the authors highlight is that both organisations rely heavily on funding from a very small number of private philanthropies. This is, as the authors argue, a risk in terms of future resource mobilization, while it also raises questions about accountability, and possible donor influence and lack of monitoring and evaluation of tobacco control initiatives.

 Although the article is indeed very insightful and well written, there are a few issues that the authors could perhaps flesh out a bit more. This could be done either in a revised version of this article or in any future work related to this theme.

 The authors are right to suggest that what undermines effective tobacco control are issues like “the segmented nature of global health institutions and government ministries,” as well “global regime complexity” and litigation by tobacco firms, yet more could be said about this and how these issues are related. What does the regime complex in tobacco control look like, why is it so problematic and there ways to solve it? To this end, the authors could perhaps provide a slightly more in depth discussion on the role of other IOs/regional (economic) organisations in tobacco control, as well as the role of national governments (i.e. the members of these subnational organisations). The authors could also draw on literature on regime complexity in other policy areas. Particularly interesting is the literature on the climate change debate in this regard ^1^.

 What is more, it would be interesting to say more about the exact implications of regime complexity for the political role and influence of tobacco companies. The key question is, as Alter and Meunier ^2^ put it, whether and how international regime complexity impacts “decision-making and political strategies, as well as empower some actors and interest groups.” That is, regime complexity provides internationally operating firms with ample opportunities to engage in forum shopping and, as such, target those institutions that are most favourably disposed towards their policy preferences. Research has shown that tobacco companies use the WTO Dispute Settlement System ^3^ and Investor-State Dispute Settlement Mechanisms within Bilateral Investment Treaties to challenge domestic tobacco control policies like the introduction of plain packaging ^4^. Further research is needed on how business-government relations are affected by the trans-nationalisation of tobacco firms and the institutional complexity of tobacco control governance.

 Finally and related, the authors briefly mention the tension between trade and health law and this issue could be unpacked further as well. During the FCTC negotiations a compromise was reached whereby no explicit reference was made to the relationship between tobacco control and international trade law. As a result, doubts have been raised as to the likely outcomes of disputes involving a conflict between trade and health concerns ^5^^-^^7^. In a recent ruling in a dispute concerning tobacco products within the WTO, where several countries complained about the adoption of plain packaging (PP) requirements by Australia, the WTO Panel ruled in favour of the defendant (Australia) and argued that the public health measure taken (i.e. the introduction of plain packaging) was in fact not inconsistent with trade law (see: https://www.wto.org/english/tratop_e/dispu_e/cases_e/ds467_e.htm).

I have read this submission. I believe that I have an appropriate level of expertise to confirm that it is of an acceptable scientific standard.

## References

[1] KeohaneRVictorD: The Regime Complex for Climate Change. *Perspectives on Politics*.2011;9(01) : 10.1017/S1537592710004068 7-23 10.1017/S1537592710004068

[2] AlterKMeunierS: The Politics of International Regime Complexity. *Perspectives on Politics*.2009;7(01) : 10.1017/S1537592709090033 13-24 10.1017/S1537592709090033

[3] CurranLEckhardtJ: Smoke screen? The globalization of production, transnational lobbying and the international political economy of plain tobacco packaging. *Rev Int Polit Econ*.2017;24(1) : 10.1080/09692290.2016.1269658 87-118 10.1080/09692290.2016.1269658 28630533PMC5473340

[4] HawkinsBHoldenC: A Corporate Veto on Health Policy? Global Constitutionalism and Investor-State Dispute Settlement. *J Health Polit Policy Law*.2016;41(5) : 10.1215/03616878-3632203 969-95 10.1215/03616878-3632203 27256810PMC5040601

[5] MamuduHMHammondRGlantzSA: International trade versus public health during the FCTC negotiations, 1999-2003. *Tob Control*.2011;20(1) : 10.1136/tc.2009.035352 e3 10.1136/tc.2009.035352 20943828PMC3089441

[6] McGradyB: Confronting the Tobacco Epidemic in a New Era of Trade and Investment Liberalization. *World Health Organization*.2012;

[7] EckhardtJHoldenCCallardCD: Tobacco control and the World Trade Organization: mapping member states' positions after the framework convention on tobacco control. *Tob Control*.25(6) : 10.1136/tobaccocontrol-2015-052486 692-698 10.1136/tobaccocontrol-2015-052486 26585705PMC4891294

# Peer review: Referee response for version 1

**DOI:** 10.21956/wellcomeopenres.15626.r32904

**Published:** 2018-05-24

**Authors:** Prabhat Jha

**Affiliations:** 1 St Michael's Hospital, University of Toronto, Toronto, Ontario, M5C 1N8, Canada

On current smoking patterns, about 1 billion smokers will be killed by their addiction in the 21 ^st^ century, as opposed to “only” 0.1 billion in the 20 ^th^ century.  Reductions in premature deaths from tobacco consumption are possible in every country, particularly with significant increases in tobacco excise taxation ^1^.

 Tobacco control involves ministries of finance, as well as vested interests in agriculture, commerce and trade, it is unusual from most health challenges that are normally within the purview of health ministries. Thus, understanding the global response to the tobacco epidemic is crucial, and given the multitude of sectors, is complex. Devi Sridhar and her team have made an important contribution to understanding the global architecture for tobacco control ^2^.  In this review, Mukaigawara and colleagues review the major periods of global tobacco control that started first with attention by the World Health Organization (WHO) and then the World Bank, and more recently by private philanthropies.

 Their review is serious, scholarly and sobering.  It points to the obvious disconnect between the huge, and largely avoidable burden from tobacco, and its response.  Tobacco taxation is, arguably, the single most cost-effective intervention for health worldwide ^3^. Yet it continues to be vastly underused. Why? The obvious reason is the enormous profitability for tobacco dealers, who earn approximately $50 billion of profit every year from cigarette sales, or roughly about $10,000 per tobacco death ^1^. The second reason, articulated nicely in this review, is that the international architecture for tobacco control has been less than optimal.  The alignment of financial and development interests with tobacco control demands a much stronger role for the World Bank and the International Monetary Fund, as key counterparts to ministries of Finance.

 The World Bank made substantial progress during its phase when it produced the widely-influential “Curbing the Epidemic” report ( ^4^; full disclosure- I led that report, and organized its funding and implementation while a Senior Health Specialist at the World Bank).  Since then, the track record has been uneven.  However in the last three years, Patricio Marquez and team have substantially reinvigorated the Bank’s response and produced an important report ^5^ that calls for a major scale of big, fast tax increases so as to change future smoking behaviour.

 WHO estimates that over the last few years, about 100 countries raised excise taxes, and that the proportion of the world now covered by some form of tobacco control has increased substantially ^6^. This is welcome news, but marked increases in adult cessation (which is the only practicable way to avoid much of the tobacco deaths prior to 2050) would need large tax hikes paired with strategies to prevent downward substitution.

 What then for the future of global tobacco control?  First, as with most health successes, country ownership is key to fund and to support tobacco control.  Here, the recent evidence that strongly links tobacco excise increases to reductions in poverty (mostly from avoided treatment cost for tobacco-attributable diseases; ^7^) is an important milestone that may increase country ownership.  Second, the forthcoming Tax Force on Fiscal Policies for Health, chaired by Larry Summers and Michael Bloomberg might provide important spur for governments to signals future price hike expectations to smokers.  Indeed, France was able to halve consumption per adult in about 15 years versus the 30 years it took to halve consumption in the United States by using large excise taxes and announcing these in advance to the public ^1^.  Today French ex-smoking prevalence among middle age adults is well above the European average.

 Mukaigawara and colleagues warn, appropriately, about the undue reliance on private philanthropic funding for tobacco control.  A small criticism of an otherwise fine paper is that they did not spend sufficient time discussing research innovations for tobacco control (such as better and more local epidemiological and economic research) or new tools (such as is now appearing with use of plain packaging; ^3^). Indeed, a useful trajectory of philanthropic research funding would be to move away from implementation to a much deeper investment in creating new tools, and evidence-driven networks that could support countries to raise taxes substantially.

 Large tax hikes might well avoid about 200 million deaths over the next few decades ^1^ and are the only practicable way to achieve the United Nations global goals of reducing deaths from chronic disease by 2030 ^7^.

I have read this submission. I believe that I have an appropriate level of expertise to confirm that it is of an acceptable scientific standard.

## References

[1] JhaPPetoR: Global effects of smoking, of quitting, and of taxing tobacco. *N Engl J Med*.2014;370(1) : 10.1056/NEJMra1308383 60-8 10.1056/NEJMra1308383 24382066

[2] MukaigawaraMWintersJFernandesGSridharD: Balancing science and political economy: Tobacco control and global health. *Wellcome Open Research*.2018;3: 10.12688/wellcomeopenres.14362.1 10.12688/wellcomeopenres.14362.1 30191182PMC6097416

[3] JhaPMacLennanMChaloupkaFJYurekliARamasundarahettigeCPalipudiKZatońksiWAsmaSGuptaPC: Global Hazards of Tobacco and the Benefits of Smoking Cessation and Tobacco Taxes. *Gelband H, Jha P, Sankaranarayanan R, Horton S, editors.Cancer: Disease Control Priorities, Third Edition (Volume 3). Washington (DC): The International Bank for Reconstruction and Development / The World Bank; 2015 Nov. Chapter 10.*.26913345

[4] JhaP: Curbing the epidemic - governments and the economics of tobacco control(English). Development in practice. *The World Bank, Washington, DC*.1999;

[5] MarquezPMoreno-DodsonB(ed): Tobacco Tax Reform at the Crossroads of Health and Development : A Multisectoral Perspective. *Washington DC. World Bank*.2017;

[6] WHO report on the global tobacco epidemic 2017. *World Health Organization*.2017;

[7] Global Tobacco Economics Consortium: The health, poverty, and financial consequences of a cigarette price increase among 500 million male smokers in 13 middle income countries: compartmental model study. *BMJ*.2018;361:k1162 2964309610.1136/bmj.k1162PMC5894369
